# The Benslimane's Artistic Model for Leg Beauty

**DOI:** 10.1007/s00266-012-9886-1

**Published:** 2012-04-26

**Authors:** Fahd Benslimane

**Affiliations:** Clinique Benslimane, 7 rue Ahmed Annaciri, Palmier , 20100 Casablanca, Morocco

**Keywords:** Legs beauty, Calves beauty, Ankles beauty, Models, Leonard de Vinci, Golden ratio, Divine proportions

## Abstract

**Background:**

In 2000, the author started observing legs considered to be attractive. The goal was to have an ideal aesthetic model and compare the disparity between this model and a patient’s reality. This could prove helpful during leg sculpturing to get closer to this ideal. Postoperatively, the result could then be compared to the ideal curves of the model legs and any remaining deviations from the ideal curves could be pointed out and eventually corrected in a second session. The lack of anthropometric studies of legs from the knee to the ankle led the author to select and study attractive legs to find out the common denominators of their beauty.

**Method:**

The study consisted in analyzing the features that make legs look attractive. The legs of models in magazines were scanned and inserted into a PowerPoint program. The legs of live models, Barbie dolls, and athletes were photographed. Artistic drawings by Leonardo da Vinci were reviewed and Greek sculptures studied. Sculptures from the National Archaeological Museum of Athens were photographed and included in the PowerPoint program.

**Results and Conclusion:**

This study shows that the first criterion for beautiful legs is the straightness of the leg column. Not a single attractive leg was found to deviate from the vertical, and each was in absolute continuity with the thigh. The second criterion is the similarity of curve distribution and progression from knee to ankle.

**Level of Evidence V:**

This journal requires that authors assign a level of evidence to each article. For a full description of these Evidence-Based Medicine ratings, please refer to the Table of Contents or the online Instructions to Authors at www.springer.com/00266.

## Introduction

**Fig. 1:**
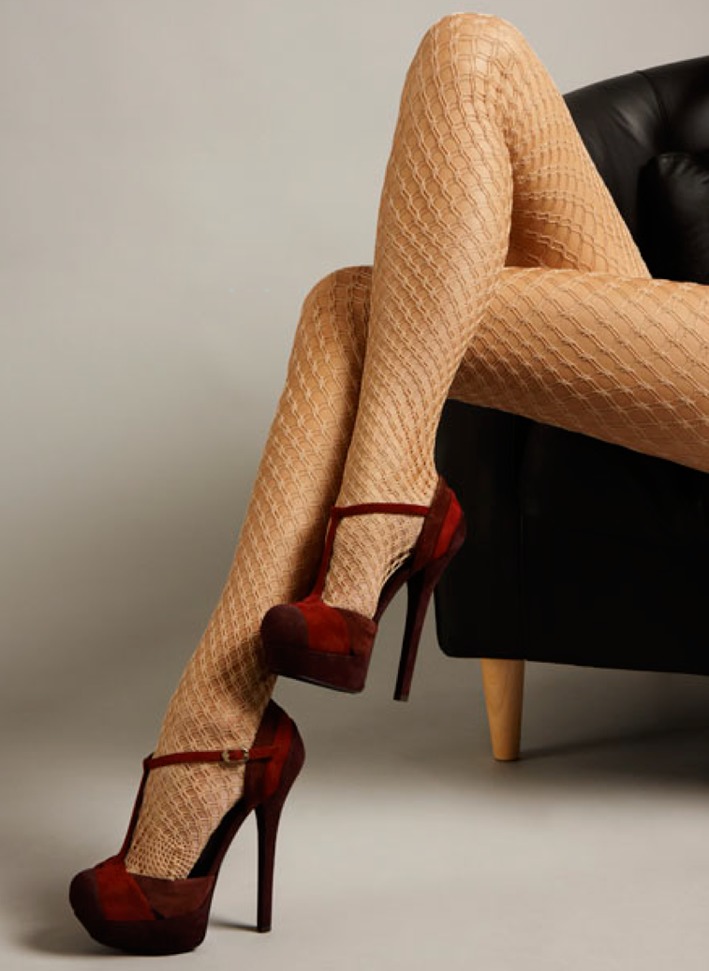
With changes in women’s fashion, legs became an important element of seduction

Over the last 50 years, with the changes in women’s fashion, legs have become an important element of seduction (Fig. 1). It is remarkable, however, that cosmetic surgery of the legs is rarely the subject of the media. The slow development of cosmetic surgery of the legs and ankles can be attributed to the fear of the frequent complications. Meanwhile, no workable aesthetic model of the leg is available.

Indeed, when slimming down of the ankles is considered, liposuction is performed in a circular fashion. The upper limits of the fat compartments to be aspirated are the lower edge of the medial and lateral gastrocnemius muscles [1]. Thus, the volume is reduced medially and laterally without considering any aesthetic ideal: do ankles have the same concavity medially as laterally? Do the lower concavities of the legs (ankles) extend cephalad in the same fashion medially as laterally? The same question applies for the calves: When enhancing or reducing the volume of the calves, should it be done in a symmetric fashion? Is the medial and lateral convexity of the calves symmetric?

In this study the author analyzes legs that are considered the most attractive in the Western world. Legs of models from Body Part Models, Inc. (http://www.bodypartmodels.com) based in Los Angeles are studied as well as legs of models in Ukraine and France. The evaluation of legs’ curves, their shape, and succession shows that there is a common denominator among all attractive legs. The Golden Ratio as applied to the legs in Leonardo da Vinci’s drawings is discussed.

## Method

Three groups of models were analyzed: models from different ethnic groups, Barbie dolls, and athletes. The author also analyzed two groups of artistic models: drawings by Leonardo da Vinci (the Vitruvian Man) and Greek sculptures (photographs taken at the National Archeology Museum of Athens).

### Models, Barbie Dolls, and Athletes

**Fig. 2:**
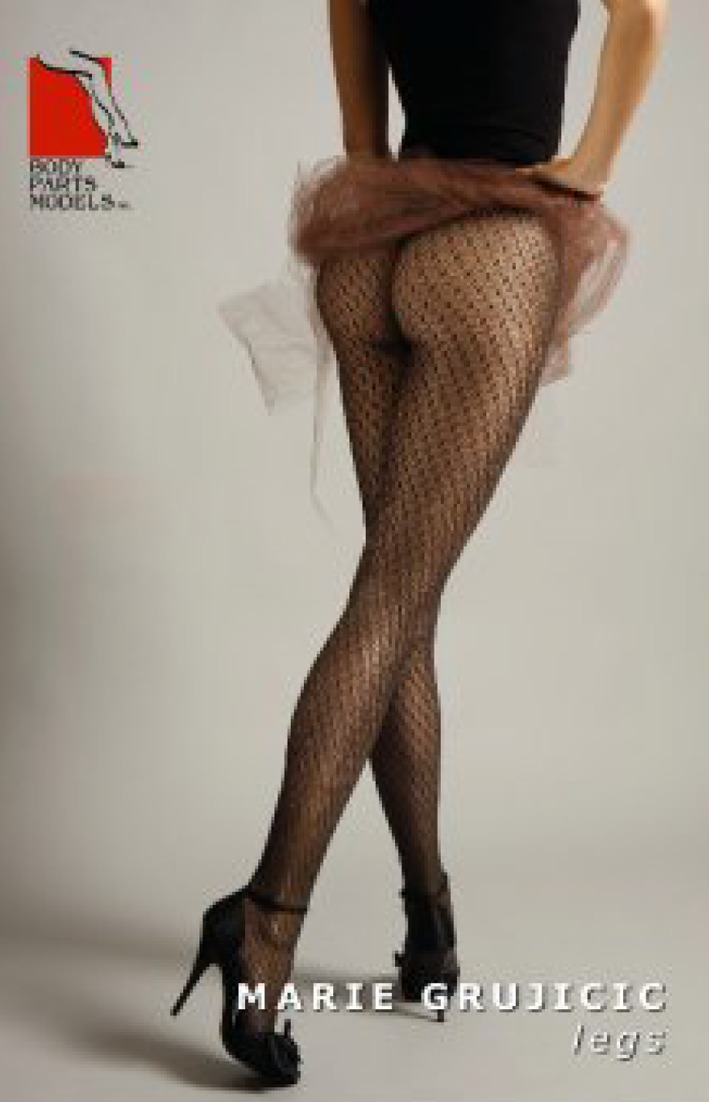
Importance of studying models’ legs considered as an ideal of beauty. The legs of Marie Delage Grujicic, a model for Body Parts Models, Inc., in Los Angeles, CA (www.bodypartsmodels.com)

A total of 556 photographs of legs found in fashion and health magazines were scanned and inserted into a PowerPoint program. Other photographs were taken of 39 live models in Morocco, France, Ukraine, Hungary, and the US. It was important for the author to include the study of models’ legs considered as an ideal of beauty. The legs of models who work as “body doubles” in the film industry were studied. Thus, the legs of Marie Delage Grujicic, a model for a Body Part Models in Los Angeles (www.bodypartsmodels.com), were examined and photographed (Fig. 2). All legs were observed from the anteroposterior view, posteroanterior view, oblique view, and lateral view.

**Fig. 3:**
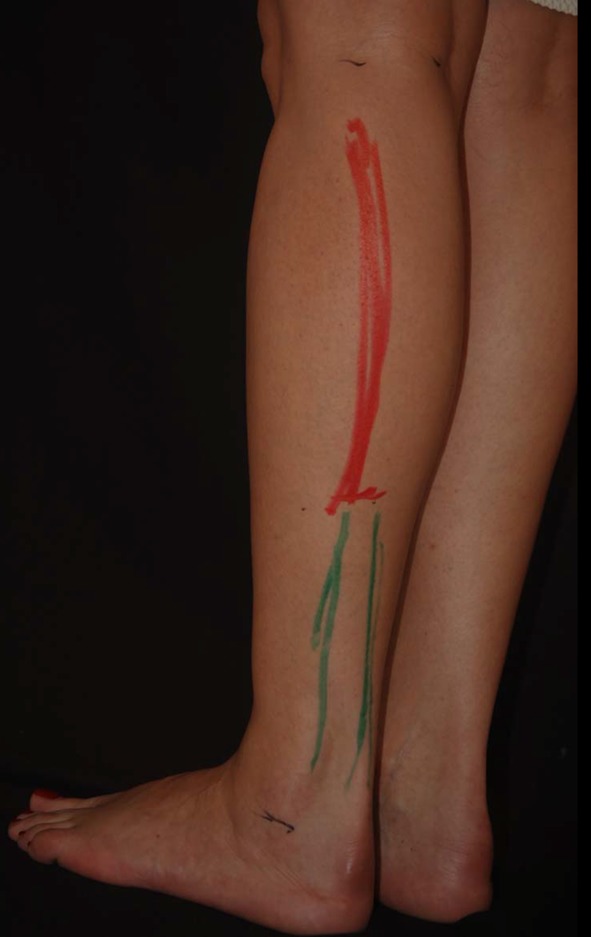
Marking of the convexities and concavities were made on some of the models with different colors

One of the most important inclusion criteria was to be able to take photographs at the exact horizontal level of the legs, i.e., not from a superior–inferior perspective as we often see in published articles. To this purpose, the models were asked to stand on a black support of 50-cm height (20 in.). The photographs were taken with the camera at the level of the midportion of the legs (between the knee and the ankle), 2 m away from the model. Other photographs were taken at the level of the knee to show the entire lower limb. All photographs were taken with a uniform black background. Photographs were taken with the feet together and apart. On some of the models, convexities and concavities were marked with different color markers (Fig. 3).

**Fig. 4:**
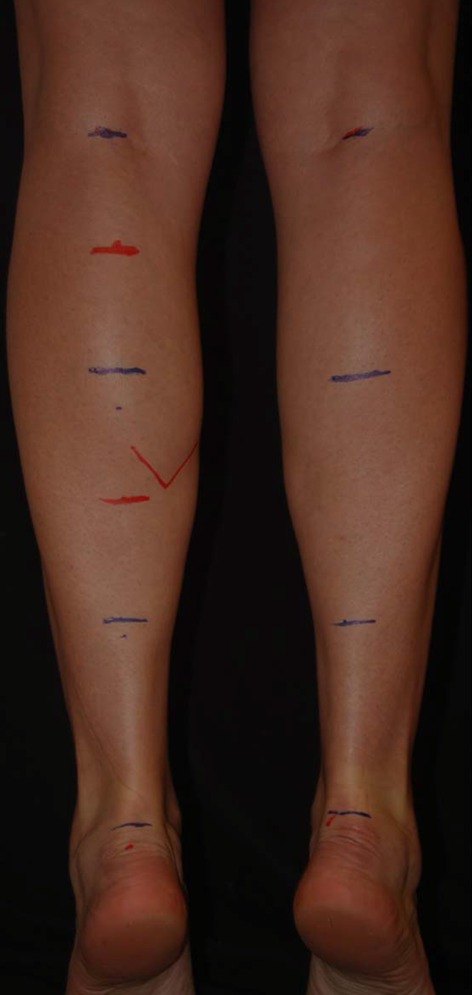
The upper limit of the leg was defined as being the midpoint of the popliteal fold and the lower edge as the lateral malleolus

The photos were inserted into the PowerPoint program. The author analyzed the photos on the anteroposterior and posteroanterior views as well as on the lateral view. The upper limit of the leg was defined as the midpoint of the popliteal fold and the lower limit was the lateral malleolus (Fig. 4).

**Fig. 5:**
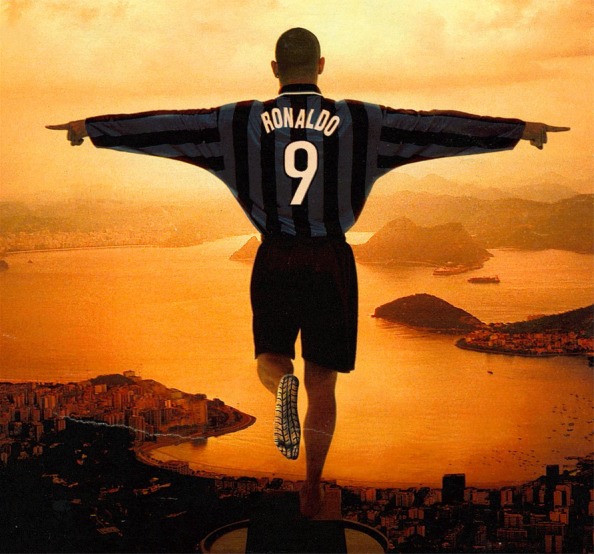
The legs of the famous Brazilian football player Ronaldo were also analyzed

As we can draw no conclusion from the study of a single group (models), the author included a completely different group in the study: legs of athletes. The legs of the famous Brazilian football player Ronaldo were also analyzed (Fig. 5).

**Fig. 6:**
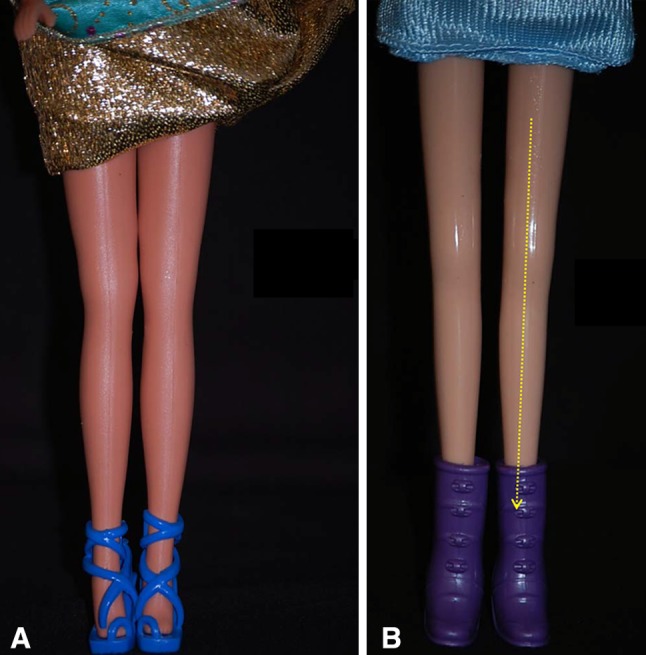
**a**, **b** Importance of studying the legs of Barbie dolls. Since the Barbie doll’s introduction in 1959, dolls stopped being simple toys and became models for young children

Finally, it was important to analyze the legs of Barbie dolls (Fig. 6a, b). Indeed, since its introduction in 1959, the Barbie doll became for the first time a model for young children, and sometimes even for adults.

### Anthropomorphic Analysis of the Legs: The Mechanical Axis of the Lower Limbs

**Fig. 7:**
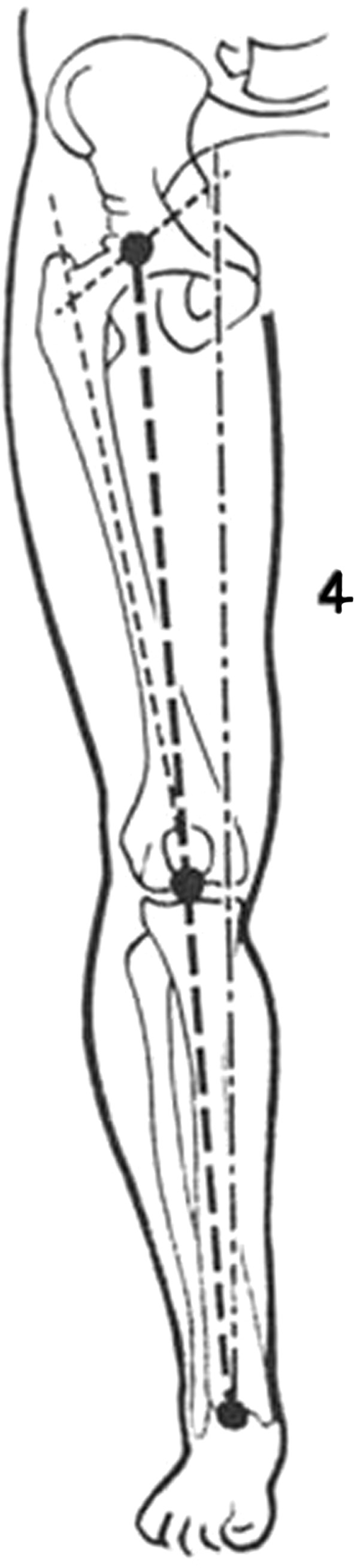
The mechanical axis of the lower limbs is defined as a straight line going through the middle of the knee joint, the femoral head, and the middle of the ankle joint

**Fig. 8:**
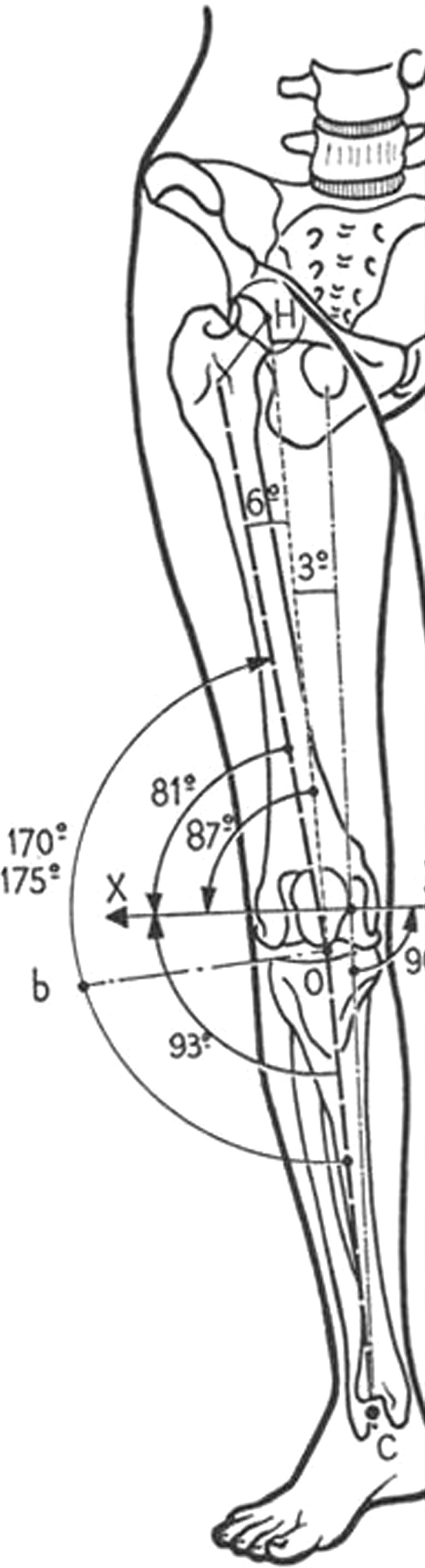
The mechanical axis deviates only 3° from the vertical axis, which confers an impression of absolute straightness

The initial observation consisted in an overview of the legs in order to analyze the axis of the legs and follow its direction. The mechanical axis of the lower limbs is defined as a straight line going through the middle of the knee joint, the femoral head, and the middle of the ankle joint (Fig. 7). This axis runs from the head of the femoral bone going slightly obliquely downwards and inwards. The mechanical axis deviates only 3° from the vertical axis (Fig. 8), which confers an impression of absolute straightness.

## Results

### Straightness of the Leg Column

**Fig. 9:**
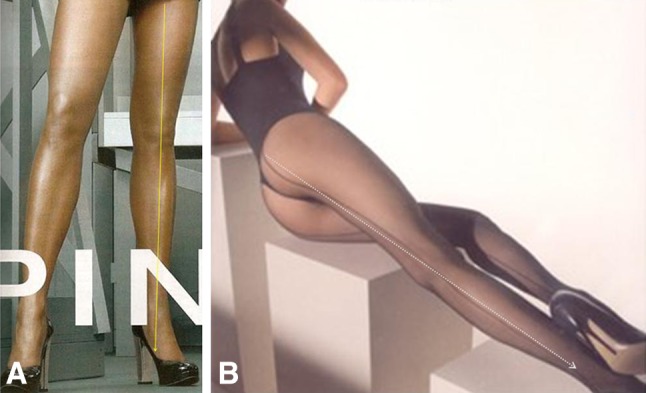
The first common denominator of all attractive legs is their straightness. Attractive legs are straight and in absolute continuity with the thighs. As soon as the leg column departs from the straight axis (genu valgum or genu varum), it deviates from our perception of beauty

**Fig. 10:**
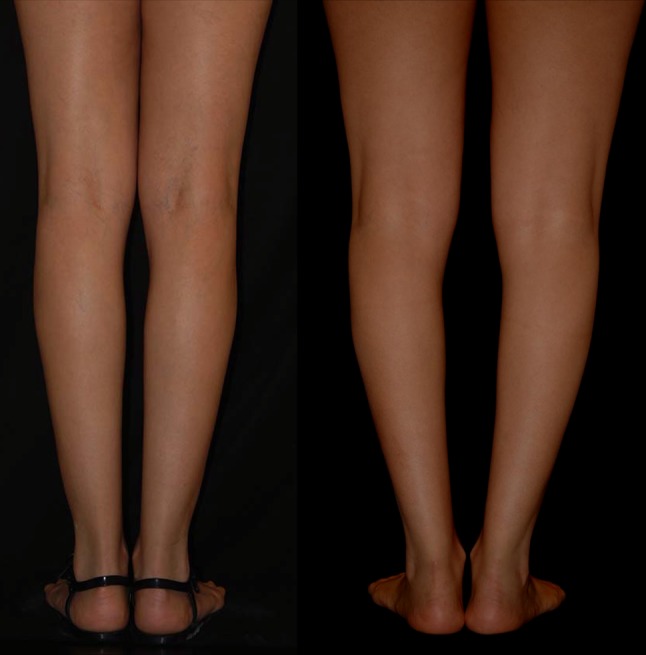
Comparison between **a** straight, shapeless legs and **b** bowed legs. As soon as the axis of the legs diverges from the straight axis, it deviates from our perception of beauty

**Fig. 11:**
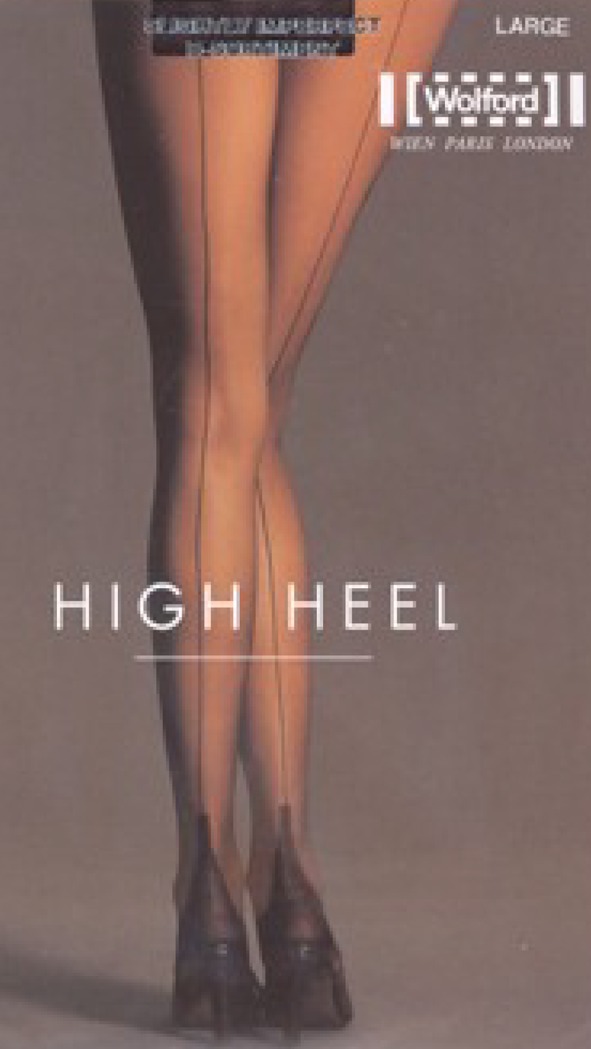
The straightness of the legs of models who advertise leg stockings and lingerie is notable

The first common denominator of all attractive legs is their straightness (Fig. 9). Attractive legs are straight and in continuity with the thighs. As soon as the leg column departs from the straight axis (genu valgum or genu varum), it deviates from our perception of beauty (Fig. 10). This is probably one of the reasons why long, straight, and slender legs are considered especially attractive. Our sense of aesthetic harmony is intensified by the blending of fragility, represented by thinness, and strength, represented by straightness. The straightness of the legs of models who advertise leg stockings and lingerie is notable (Fig. 11). Absolute straightness is fundamental to our perception of attractiveness. This straightness is found on the legs of the Barbie doll. It is interesting to note that some Barbie dolls are manufactured with extremely thin legs and no curves whatsoever and other Barbie dolls are manufactured with more voluminous, smooth, and gentle curves in their legs. However, in both cases, the leg column is absolutely straight and in sharp continuity with the thigh (Fig. 6).

**Fig. 12:**
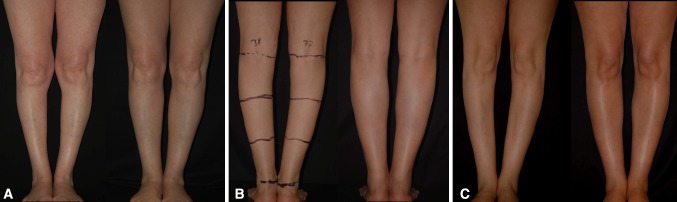
**a**–**c** The first goal when performing a leg sculpture is to give straightness to the leg column using either liposuction, microfat grafting, or both

Thus, the first goal for the author when performing a leg sculpture is to make the leg column straight, using liposuction, microfat grafting, or both (Fig. 12a–c).

### Similarity of Curve Distribution Among Attractive Legs

#### Subjective Observation

**Fig. 13:**
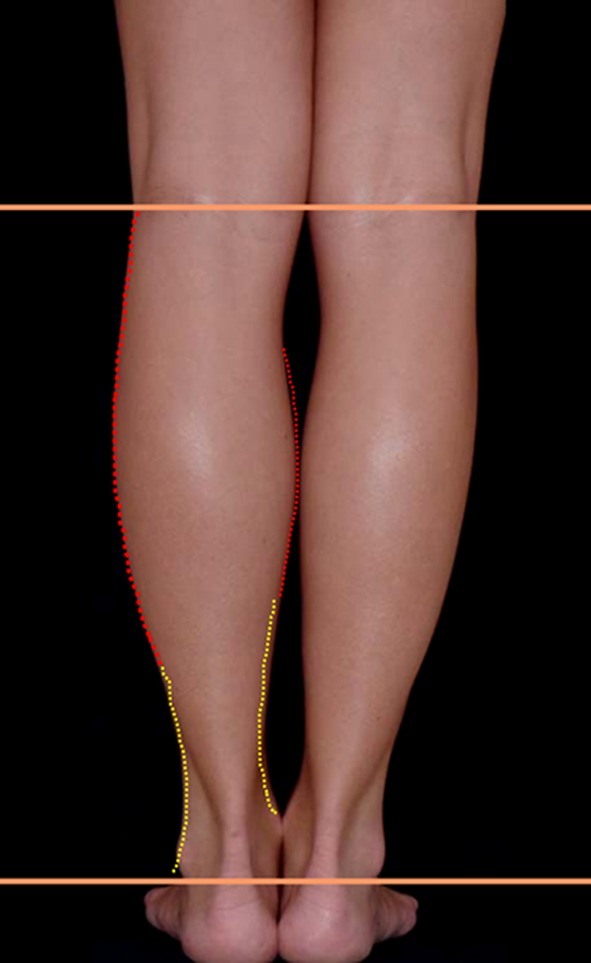
The convex and concave curves were outlined with different colors in the PowerPoint program: red for convexities and yellow for the concavities. This was done on the medial, lateral, posterior, and anterior aspect of the leg

The convex and concave curves were outlined on the legs with different colors on the PowerPoint program: red for the convexities and yellow for the concavities (Fig. 13). This was done on the medial, lateral, posterior, and anterior aspect of the leg. We found that medially the convex curve is short and very pronounced, followed by a long concavity. Laterally, the convexity is longer and smoother*.* It is followed by a gentle short concavity that ends at the external malleolus (Fig. 13).

#### Objective Scientific Analysis: Geometry

**Fig. 14:**
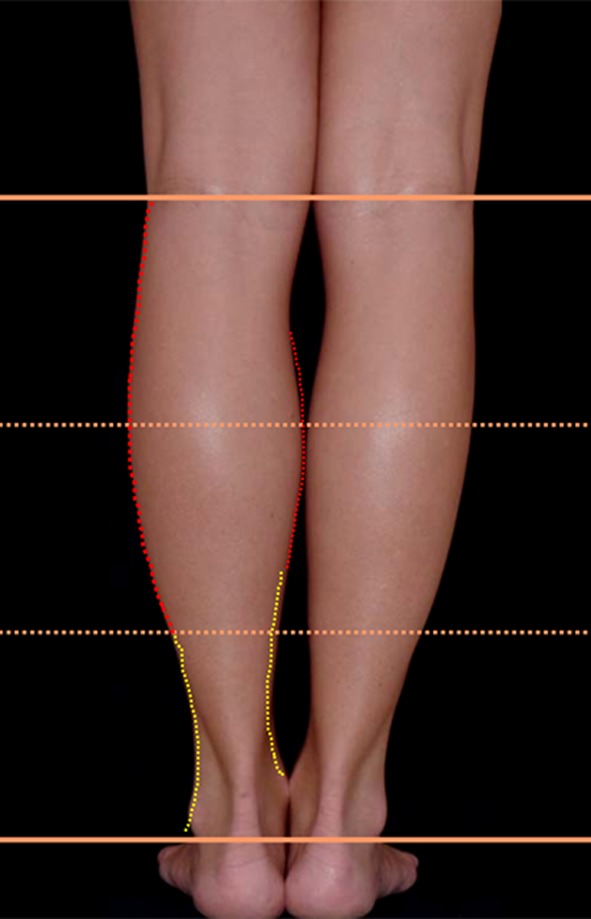
For didactic purposes, the legs are divided into thirds. The medial and lateral convexities of the legs are fundamentally asymmetric. All the models had a medial convexity that straddles the upper and middle third while the lateral convexity stretches along the upper and middle thirds

**Fig. 15:**
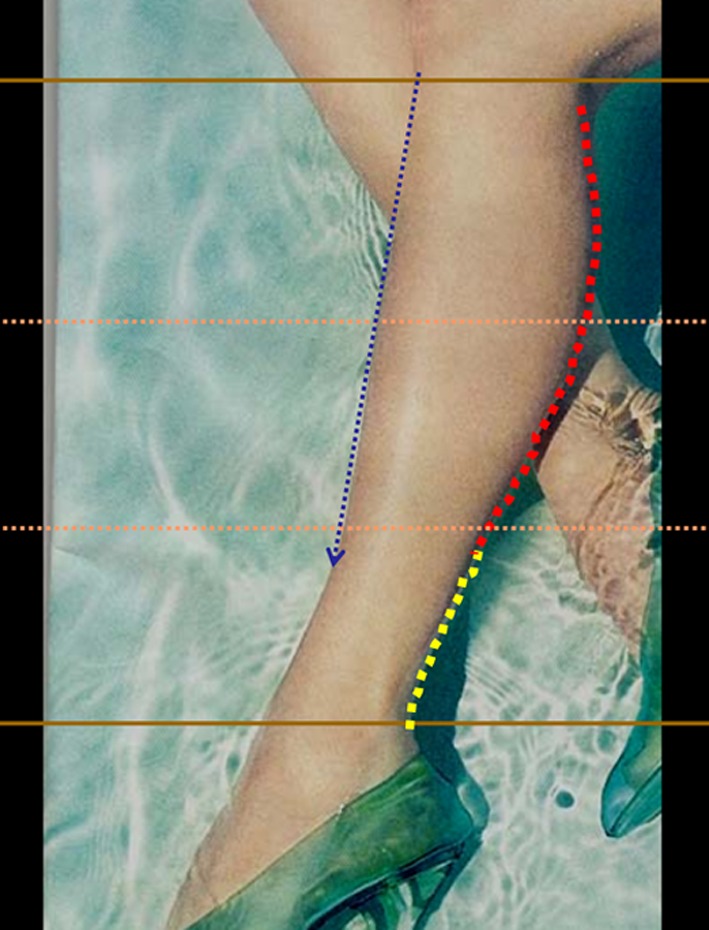
From the side view, the posterior convexity entirely occupies the upper two thirds of the leg just like in its lateral aspect. The anterior leg is almost a straight line

For didactic purposes and to move from a subjective observation to a more scientific analysis, the author called upon geometry. The legs were divided into thirds (Fig. 14). It was found that all the models have a medial convexity that straddles the upper and middle third (the lower half of the upper third and the upper half of the middle third), while the lateral convexity stretches along the upper and middle thirds (Fig. 14). Thus, the medial and lateral convexities of the legs are fundamentally asymmetric*.* From the side view, the posterior convexity occupies the upper two thirds of the leg, just like in its lateral aspect. The anterior leg is an almost straight line (Fig. 15).

**Fig. 16:**
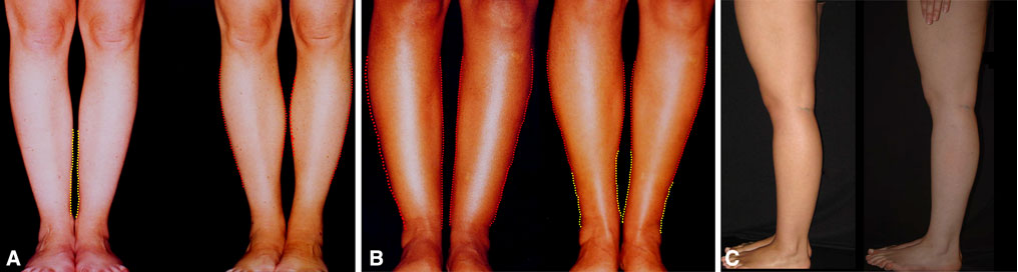
**a**–**c** The second goal when performing a leg sculpture is to reproduce, as much as possible, the delicate asymmetric curves found on models’ legs

**Fig. 17:**
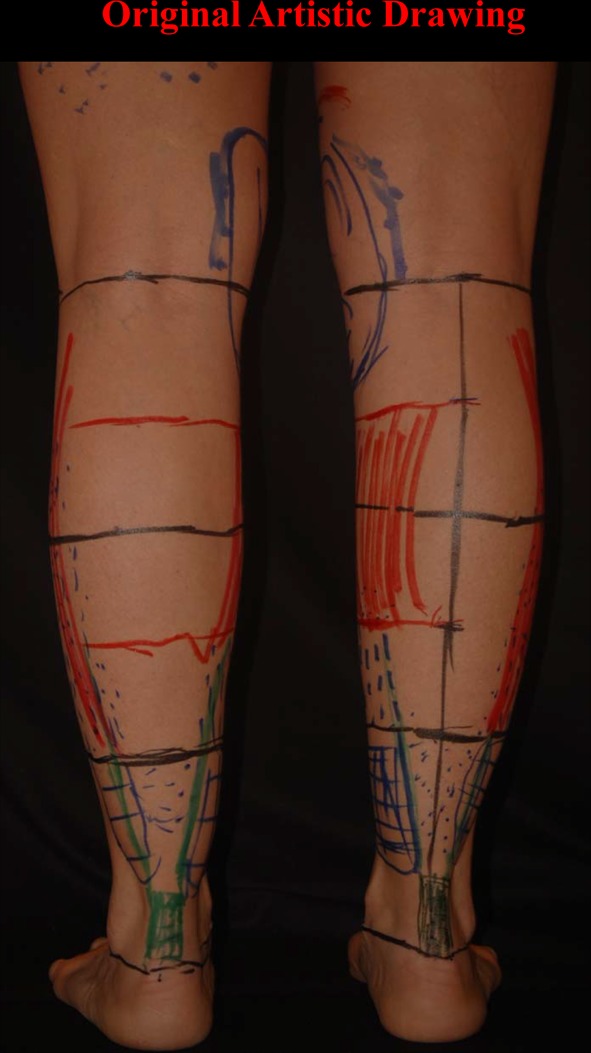
The original artistic drawing developed by the author

The second goal when performing a leg sculpture is to reproduce, as much as possible, the delicate asymmetric curves found on models’ legs (Fig. 16a–c). This is done according to the original artistic drawing developed by the author (Fig. 17).

### Artistic Drawings and Sculptures

#### Leonardo da Vinci Drawing: the Vitruvian Man

**Fig. 18:**
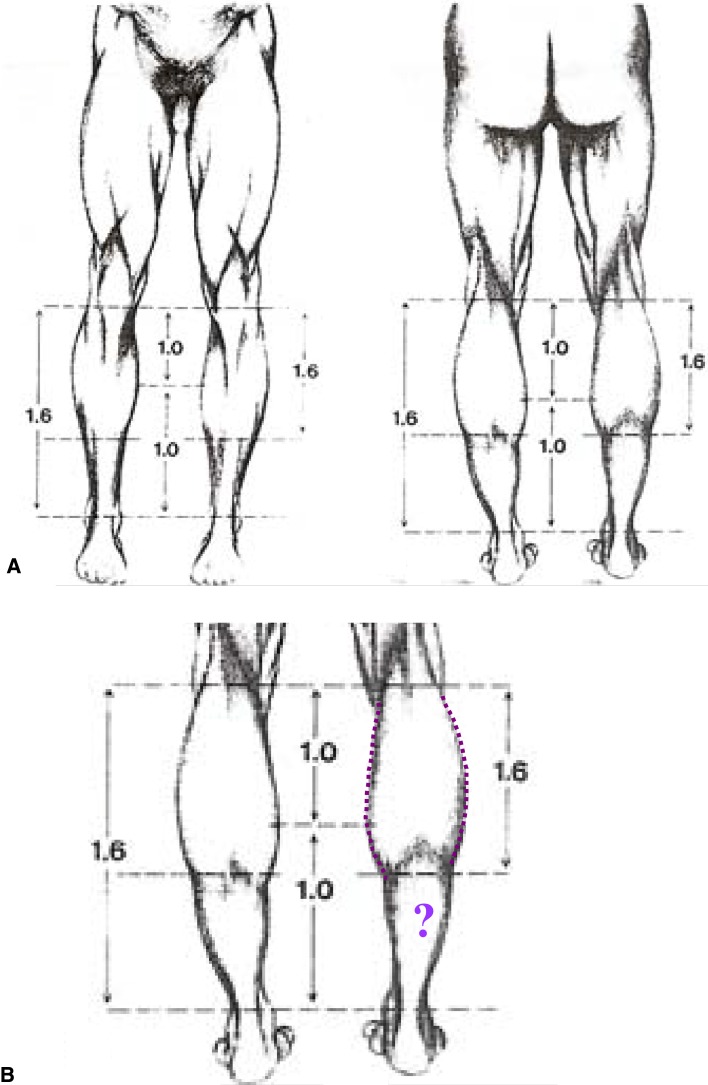
**a**, **b** The curves of the legs of the Vitruvian Man show perfect symmetry between the medial and lateral aspect of the legs. A shape like this does not exist on the legs of contemporary man

The curves of the legs of the Vitruvian Man show perfect symmetry between the medial and lateral aspects of the legs! (Fig. 18a, b). Legs shaped like this do not exist in contemporary mankind. The legs may have been drawn like this because since there were no means of locomotion in the Renaissance era, probably man’s leg muscles were more developed, resulting in such drawing. If this were the case, these curves should be found on sculptures of the pre-Renaissance era.

### Sculptures from Ancient Greece

**Fig. 19:**
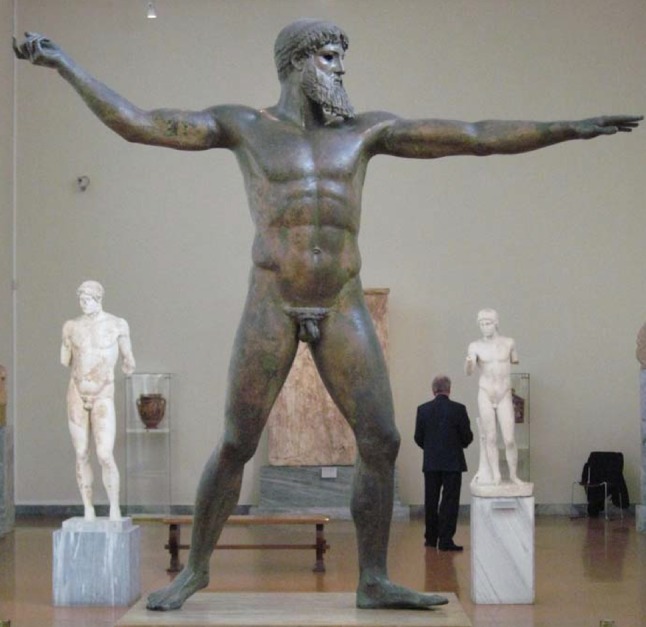
Sculpture of Zeus depicting the ideal human form of ancient times (photograph taken by the author at the National Archeology Museum, Athens)

**Fig. 20:**
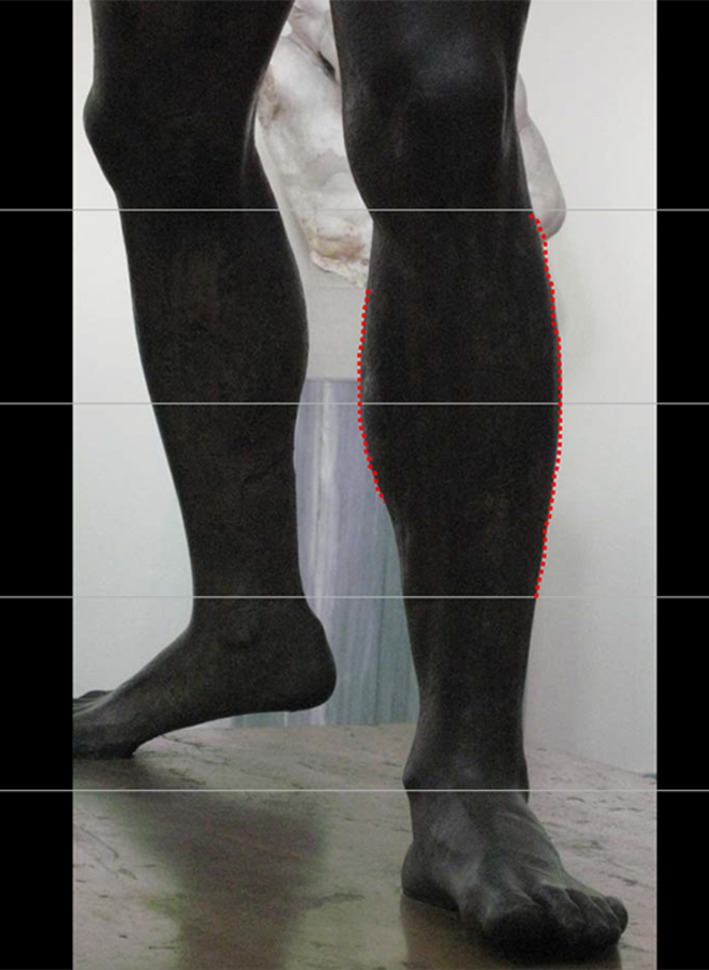
The shape and distribution of the curves of ancient legs are exactly the same as those of men and women models today (photograph taken by the author at the National Archeology Museum, Athens)

During a visit to the National Archaeology Museum of Athens, the author observed sculptures that depict the ideal human form of those ancient times (Figs. 19, 20). Some of them were dated back to 2000 BC (Zeus and Poseidon). The shape and distribution of the curves of such ancient legs are exactly the same as those of men and women models today! (Fig. 20).

How does one interpret the drawings of the great Leonardo da Vinci? Pythagoras (6 BC) was the first to argue that the principle of all things is the number. The Pythagoreans seek in the number the rule capable of limiting reality, to give order and understanding to the universe. Leonardo da Vinci adhered strongly to the Pythagorean “mathematical-aesthetic vision” of the universe. “Beauty cannot be scientific till it takes a mathematical expression” was among his favorite expressions. Can we consider that Leonardo da Vinci “adapted” his drawing of the leg in order to “make it correspond” (get in the mold) to the Golden Ratio? (Fig. 18a, b). This question will remain unanswered.

## Discussion

Illouz [1] described two fat compartments: lateral and medial. He specified that the upper limit of the fat compartment to be aspirated is the lower edge of the gastrocnemius muscle. The patient should go on tiptoe to determine the lower edge of the muscles. However, performing liposuction caudal to the lower edge of the muscles, even by a gradient, assumes that the inner and outer edges of the legs and ankles are symmetrical.

Chamosa [2, 3] described the ankle and the distal leg as a rhomboid prism with a major anteroposterior axis, four sides, and four edges. This description is correct and helps in identifying the four fat compartments on the ankle. However, it does not describe the distribution of curves of the legs from knee to ankle.

In his trek toward the “ideal beautiful normal,” Howard [4] applied the “divine proportion,” as described by Ricketts [5], to the lower extremity. However, it was applied only to the medial aspect of the calves in order to determine what he called the medial “peak.” No mention was made to the medial concavity or to the subtle lateral convex–concave curves. The lateral sweep was described as a long, easy curve that should mimic the lateral gastrocnemius muscle. One should note that the lateral easy curve does not run from the head of the peroneus to the lateral malleolus; the lateral easy convex curve turns into a slight concavity at the lower third to end up on the lateral malleolus.

**Fig. 21:**
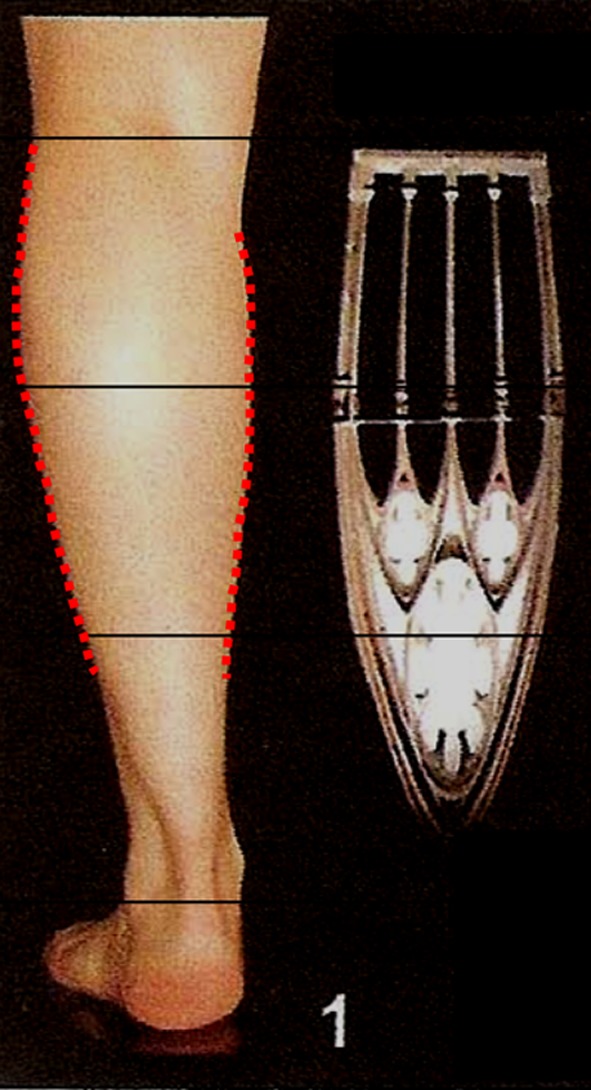
The leg taken as a model in the study by Cuenca Guerra et al. [6] does not have the form of an inverted “gothic” arc as stated in their article (reprinted with permission from [6])

Cuenca-Gerra et al. [6] have proposed a model for calf augmentation. They suggested that the two most attractive feature variables are the anteroposterior (AP) and laterolateral (LL) projections. They correctly identified the junction of the upper and middle thirds of the leg as the point of the highest AP and LL projections. They used Fibonacci’s numerical sequence to identify the ideal projection of the calf. They stated that in the posterior view, the leg has the shape of an inverted “pointed gothic arc” and that the relationship with the ankle is 1.618:1 (the divine proportion = phi) (Fig. 21). This would suggest that the lateral and medial convexities are alike, i.e., symmetrical. A quick posterior view examination of the leg clearly shows that the medial and the lateral convexities of the legs are fundamentally not symmetrical. The authors also suggested that from the lateral perspective, the leg has the shape of a half-inverted pointed arc with the convexity to the posterior side. Once again, examining the leg from the side perspective shows that the convexity in the upper two thirds inverts into a concavity in the lower third (Fig. 15). Finally, the leg used as a model in their study did not have enough convexity in its medial aspect to balance the lateral curve. In my view, the medial upper convexity followed by the lower pronounced concavity is one of the most attractive features that defines leg beauty.

Most plastic surgeons who studied leg aesthetics reference Ricketts’ article “The Biologic Significance of the Divine Proportion and Fibonacci Series” [5]. However, the article focused on facial and dental proportions but no mention was made to the legs. Moreover, the drawing used by Ricketts does not portray (illustrate) the application of the golden ratio to the lateral aspect of the legs.

Art historians as well as theorists of the Divine Numbers agree that the Golden Ratio is inherent in every work of art considered beautiful. This ratio ultimately describes the absolute and unique beauty. To understand and interpret beauty, one must return to the basics: the Pythagoras-Platonic heritage contained in the works of Euclid [7].

We are not completely sure if Pythagoras practiced geometry! In fact, all of the works attributed to him are apocryphal, though he is considered by some the inventor of Greek mathematics [8–10]. This is another myth that may have been perpetuated until the end of the fourth century BC to explain the origins.

The Pythagoreans formed a heterogeneous group, few of whom actually practiced mathematics, except for one: Archytas of Tarentum (around 430–348 BC). The Pythagoreans were especially interested in the philosophy and mysticism of mathematics. In fact, the “number” was for them a fundamental concept that could explain the world as a whole. The expression “everything is number” offers mainly a metaphysic, and the numbers are integers, whole numbers, equal to or >2. Respected art historians think that “it is simply impossible to speak of shared numbers, percentages or averages (the relation between two parts, either quantitative or qualitative) in Pythagorean or Euclidian theorems” [7].

## Conclusion

It is natural that some plastic surgeons have tried to find in numbers the secret of beauty. Thus, the Golden Ratio has been applied in this quest. However, one should not try at all costs to find mathematical rules to define facial or body beauty. The use of the Golden Ratio has been deceptive so far. The proof is that no mathematical formula has been universally accepted to define any area of the face or body by the plastic surgery community. How many plastic surgeons use the Golden Ratio in their daily practice? That said, when the most ideal and generally admired proportions of leg beauty are studied and thoroughly understood, the cosmetic surgeon may better close the gap between the patient’s reality and the ideal aesthetic model found in nature.
